# End-of-Life Decision Making in Chronic Life-Limiting Disease: A Concept Analysis and Conceptual Model

**DOI:** 10.1093/geroni/igaa057.1346

**Published:** 2020-12-16

**Authors:** Kristin Levoy, Elise Tarbi, Joseph De Santis

**Affiliations:** 1 University of Pennsylvania, Philadelphia, Pennsylvania, United States; 2 University of Miami, Coral Gables, Florida, United States

## Abstract

Most deaths from chronic life-limiting diseases are preceded by end-of-life decisions, yet patients and caregivers are ill-equipped to make them. The lack of a common vocabulary surrounding end-of-life decision making and the paucity of conceptual models that explicate its components hamper improvements in clinical practice and research. Walker and Avant’s method for concept analysis was used to investigate uses of “end-of-life decision making” in the literature in order to identify its components (antecedents, attributes, consequences), describe stakeholder roles (patients, family/caregivers, health care providers), and develop a conceptual model. An iterative search strategy resulted in 143 included sources. These encompassed 1,127,095 patients (primarily older adults), 3,384 family/caregivers, and 588 healthcare providers. Evidence revealed that end-of-life decision making is a process, not a discrete event. This begins with preparation (antecedents), which involves the designation of a decision maker and iterative patient, family/caregiver, and healthcare provider communication across the chronic illness. These preparatory processes inform end-of-life decisions, which possess three attributes: 1) serial choices in the terminal illness phase that are 2) weighed in terms of their potential outcomes 3) through patient and family/caregiver collaboration. The components impact patients’ death experiences, caregivers’ bereavement, and healthcare systems (outcomes). The resulting conceptual model highlights the larger context of preparation (beyond advance care planning) and the prominent role of caregivers in the end-of-life decision making process. Enhanced measurement must account for the dose, content, and quality of the preparation and decision components that collectively contribute to outcomes, which holds implications for practice improvements and research.

